# Laminarin-triggered defence responses are geographically dependent in natural populations of *Solanum chilense*

**DOI:** 10.1093/jxb/erad087

**Published:** 2023-03-06

**Authors:** Parvinderdeep S Kahlon, Andrea Förner, Michael Muser, Mhaned Oubounyt, Michael Gigl, Richard Hammerl, Jan Baumbach, Ralph Hückelhoven, Corinna Dawid, Remco Stam

**Affiliations:** Chair of Phytopathology, TUM School of Life Sciences, Technical University of Munich, Emil-Ramann-Str. 2, 85354, Freising, Germany; Chair of Phytopathology, TUM School of Life Sciences, Technical University of Munich, Emil-Ramann-Str. 2, 85354, Freising, Germany; Chair of Phytopathology, TUM School of Life Sciences, Technical University of Munich, Emil-Ramann-Str. 2, 85354, Freising, Germany; Research Group of Computational Systems Biology, University of Hamburg, Notkestrasse 9, 22607, Hamburg, Germany; Chair of Food Chemistry and Molecular Sensory Science, TUM School of Life Sciences, Technical University of Munich, Lise-Meitner-Str. 34, 85354 Freising, Germany; Chair of Food Chemistry and Molecular Sensory Science, TUM School of Life Sciences, Technical University of Munich, Lise-Meitner-Str. 34, 85354 Freising, Germany; Research Group of Computational Systems Biology, University of Hamburg, Notkestrasse 9, 22607, Hamburg, Germany; Computational BioMedicine lab, Institute of Mathematics and Computer Science, University of Southern Denmark, Campusvej 55, Odense, Denmark; Chair of Phytopathology, TUM School of Life Sciences, Technical University of Munich, Emil-Ramann-Str. 2, 85354, Freising, Germany; Chair of Food Chemistry and Molecular Sensory Science, TUM School of Life Sciences, Technical University of Munich, Lise-Meitner-Str. 34, 85354 Freising, Germany; Department of Phytopathology and Crop Protection, Institute for Phytopathology, Kiel University, Hermann Rodewald Str 9, 24118 Kiel, Germany; University of Ghent, Belgium

**Keywords:** Diversity, early immune response, ethylene, laminarin, phytohormones, *Phytopthora infestans*, reactive oxygen species resistance, *Solanum chilense*, tomato

## Abstract

Natural plant populations are polymorphic and show intraspecific variation in resistance properties against pathogens. The activation of the underlying defence responses can depend on variation in perception of pathogen-associated molecular patterns or elicitors. To dissect such variation, we evaluated the responses induced by laminarin (a glucan, representing an elicitor from oomycetes) in the wild tomato species *Solanum chilense* and correlated this to observed infection frequencies of *Phytophthora infestans*. We measured reactive oxygen species burst and levels of diverse phytohormones upon elicitation in 83 plants originating from nine populations. We found high diversity in basal and elicitor-induced levels of each component. Further we generated linear models to explain the observed infection frequency of *P. infestans*. The effect of individual components differed dependent on the geographical origin of the plants. We found that the resistance in the southern coastal region, but not in the other regions, was directly correlated to ethylene responses and confirmed this positive correlation using ethylene inhibition assays. Our findings reveal high diversity in the strength of defence responses within a species and the involvement of different components with a quantitatively different contribution of individual components to resistance in geographically separated populations of a wild plant species.

## Teaser

Large-scale screenings reveal geographically distinct intraspecific differences in the dominant physiological pathogen defence responses upon glucan elicitor treatment in a wild tomato species.

## Introduction

Plant defence responses against pathogens in natural populations are often polymorphic (Kahlon and Stam, 2021a). The resistance of the host can result from the major resistance proteins or from polygenic defence mechanisms against the pathogen (Vanderplank, 1963). The latter type of resistance mechanism is often considered as basal defence, whereas major gene-mediated resistance is observed as pathogen genotype dependent. Genes encoding nucleotide-binding domain leucine-rich repeat-containing proteins (NLR) are one example of major resistance genes. *NLR* genes have been studied in natural plant populations and are reported to be diverse at the genetic level (Stam *et al.*, 2019a; Van de Weyer *et al.*, 2019; Witek *et al.*, 2021). Similarly, members of the receptor-like protein (RLP) family show intraspecific variation in the presence or absence of defence responses or variability in expression patterns of these genes (Van der Hoorn *et al.*, 2001; Kruijt *et al.*, 2005; Kahlon *et al.*, 2020, Steidele and Stam, 2021). In many cases, major gene-mediated resistance is complete. By contrast, basal resistance is defined as quantitative and pathogen race non-specific (Vanderplank, 1963). This might partially be explained by its polygenic nature, and because underlying defence reactions can be activated upon exposure to elicitors or conserved pathogen-associated molecular patterns (PAMPs). Flagellin peptides (flg22, flgII-28) and chitin have been the dominant PAMPs for studies on resistance mechanisms in plants against pathogens from bacterial and fungal lineages, respectively. Many additional PAMPs have been identified, e.g. VmE02 homologues, produced by various fungi and oomycetes, can trigger immunity response in *Nicotiana benthamiana* (Nie *et al.*, 2019), and peptide elicitor fractions from several *Fusarium* spp. activate basal defence mechanisms in Arabidopsis (Coleman *et al.*, 2021). One other example is laminarin, which is perceived in different plant species like *Nicotiana tabacum* (Klarzynski *et al.*, 2000; Meénard *et al.*, 2004), grapevine (*Vitis vinifera*; Aziz *et al.*, 2003), Arabidopsis (Meénard *et al.*, 2004), tea (*Camellia sinensis*; Xin *et al.*, 2019), *Nicotiana benthamiana*, *Hordeum vulgare*, *Brachypodium distachyon* (Wanke *et al.*, 2020), and olive (*Olea europaea*; Tziros *et al.*, 2021). Laminarin is an oligomeric β-1,3-glucan with β-1,6-glucan branches. β-1,3- and β-1,6-glucan are the major components of the oomycete cell wall (Aronson *et al.*, 1967) and may induce defence responses similar to those provoked by elicitors from the oomycete lineage.

Several molecular mechanisms have been shown to play an important role in basal defence responses. Many of these responses happen shortly upon contact with the pathogen. In some plant–pathogen interactions, basal immune responses can be quantified within the first minutes of the interaction with the pathogen or pathogen-specific molecules by measuring reactive oxygen species (ROS) production (Torres *et al.*, 2006). Fine-tuning in the production of ROS is one important cue toward activating resistance mechanisms that lead to the production of various phytohormones or activation of downstream defence regulators (Ramirez-Prado *et al.*, 2018). Roberts *et al.* (2019) showed the amount of ROS production varied when different tomato (*Solanum lycopersicum*) accessions were treated with flagellin peptides (flg22, flgII-28). Besides the production of ROS, phytohormones present or induced in the plant can greatly influence the resistance outcome. A higher level of salicylic acid (SA) is important in activating defence response in cultivated tomato leaves against *P. infestans* (Jeun *et al.*, 2000). Genes involved in ethylene (ET) and SA pathways are important in *N. benthiamana* after infection with *P. infestans* (Takemoto *et al.*, 2018). A study on potato shows that upon infection with *P. infestans*, large sets of genes are up-regulated at multiple time points post-inoculation. These included key marker genes involved in the jasmonic acid (JA) signalling pathway and genes involved in primary and secondary metabolite pathways (Tian *et al.*, 2006). In cultivated tomato the negative role of abscisic acid (ABA) in resistance against *Botrytis cinerea* is regulated by repressing SA signalling (Audenaert *et al.*, 2002), whereas resistance against *Alternaria solani* is enhanced upon exogenous ABA application through defence-related gene activation and defence-related enzymatic activity of phenylalanine ammonia-lyase (PAL), polyphenol oxidase, and peroxidase (Song *et al.*, 2011). Exogenous application of indoleacetic acid (IAA) in the soil resulted in *Fusarium oxysporum lycopersici* disease suppression in tomato plants (Sharaf and Farang, 2004).

The different components of the phytohormone signalling pathways can have positive or negative feedback effects on each other and thus form a complex interactive signalling network (Pieterse *et al.*, 2009). These complex network topologies generated the hypothesis that one or multiple components involved in the resistance need to pass a certain threshold in order for defence to be functional (Windram and Denbi, 2015). Which factors are dominant might differ dependent on the origin of a plant or population and the pathogen in question.

Such differences in dominant effective defence responses in different Arabidopsis accessions are shown by Velásquez *et al.* (2017) against the bacterial pathogen *Pseudomonas syringae* pv. tomato (*Pst*) DC3000. They found that *Pst* DC3000 resistance was mainly mediated by an increased level of the phytohormone SA in three accessions, whereas other mechanisms were dominant in 11 other resistant accessions. Another study showed that in six Arabidopsis accessions large variation in JA- and SA-associated basal resistance resulted in varied resistance against the nectrotrophic pathogen *Plectosphaerella cucumerine* and the hemibiotrophic bacterium *P. syringae*, respectively (Ahmad *et al.*, 2011).

We used a wild tomato species, *Solanum chilense*, to elucidate molecular cues behind the diversity in resistance against the oomycete *P. infestans* (Stam *et al.*, 2017; Kahlon *et al.*, 2021a). *Solanum chilense* is a suitable organism with which to study the variation of molecular responses associated with basal defence mechanisms. Populations of *S. chilense* are geographically structured in four distinct groups based on genomic studies (Böndel *et al.*, 2015; Stam *et al.*, 2019a). The two southern groups are recent expansions of the species, are genetically more divergent, and might be developing into new subspecies (Raduski and Igić, 2021). Thus the system provides a strong genetic structure. Previously, we found variation in defence responses against the apoplastic fungal leaf pathogen *Cladosporium fulvum* (syn. *Fulvia fulva*, *Passalora fulva*), with complete loss of pathogenic protein recognition in plants from the southern groups (Kahlon *et al.*, 2020). In order to assess phenotypic variation in resistance, we also quantified the number of successful infection events in *S. chilense* plants after drop inoculation with various other leaf pathogens. We showed clear differences in the frequency of successful infections after inoculation of three common filamentous pathogens in different *S. chilense* populations (Stam *et al.*, 2017). In a recent report (Kahlon *et al.*, 2021a) we showed that differences in *P. infestans* resistance between the geographically distinct populations of *S. chilense* are predominantly driven by the host genotype and can likely be attributed to differences in basal resistance, rather than isolate-specific resistance.

Here, we aim to dissect the various possible immune responses in *S. chilense* populations. We specifically investigate the early basal defence responses in *S. chilense* upon challenge with the non-specific elicitor laminarin. We confirm that laminarin elicits a subset of defence responses triggered by *P. infestans*, we report high diversity in several key regulators of basal immune responses in *S. chilense* within and between populations of the species within the first hours of infection, and we assess their individual and joint effect on the interaction outcome, by comparing these results with our previously published infection data (Kahlon *et al.*, 2021a).

## Materials and methods

### Plants material used and maintained

We used 83 plants of *S. chilense* originating from nine populations (accessions) (8–10 plants each): LA1958, LA1963, LA2747, LA2932, LA3111, LA3786, LA4107, LA4117A, and LA4330. The seeds of these populations were procured from the C. M. Rick Tomato Genetics Resource Center of the University of California, Davis (TGRC UC-Davis, http://tgrc.ucdavis.edu/) where the populations were originally collected as a random collection of seeds from the wild populations and are now maintained and propagated at the TGRC to maintain genetic diversity. Procured seeds were sown and plants were maintained in controlled greenhouse conditions (16 h light and 24 °C temperature in the daytime and 18–20 °C at night) at Technical University of Munich’s plant technology centre. Each plant used in this study was at least a year old. Plants were maintained throughout the experiments by cutting them every 2 weeks. Each population used in this study represents one of four geographical locations of the species habitat, and they were originally collected during different years from wild populations. Each individual plant within a population is genetically unique.

### Evaluation of laminarin potential to activate early immune responses similar to *P. infestans* using 3ʹ RNAseq

We selected the central population, LA3111, to evaluate differentially expressed genes in the transcriptome upon challenging with *P. infestans* Pi100 (3000 sporongia ml^−1^) and laminarin (1 mg ml^−1^, Sigma-Aldrich) treatment (using spray inoculation). We used the LA3111 population because the reference genome of *S. chilense* was generated from an individual from this population (Stam *et al.*, 2019b). To measure the general defence responses in the population, the experiment was done on nine plants and all plants were pooled per treatment for the RNA extraction. Detached leaves were kept upside down in plastic boxes containing wet tissue beds and treated with water, laminarin, or *P. infestans*. The boxes were kept at 18–20°C for 3 h and samples were taken and snap-frozen in liquid nitrogen. RNA was isolated using the Qiagen RNeasy plant mini kit according to the instruction manual. Each treatment consisted of pooled samples of nine plants and four technical replicates of each treatment.

3ʹ-RNA libraries were prepared according to the manufacturer’s protocol using the QuantSeq 3ʹmRNA-Seq Library Prep Kit (Lexogen, Vienna, Austria). Sequencing was performed on a HiSeq2500 (Illumina, San Diego, CA, USA) with single-end 100 bp reads using Rapid SBS v2 chemistry. The raw sequencing reads in FastQ format were trimmed to remove adapter sequences using Trimmomatic v0.39 (Bolger *et al.*, 2014). The reads were quality filtered and trimmed using the following settings: LEADING:3, SLIDINGWINDOW:4:15, MINLEN:40. HISAT2 (Kim *et al.*, 2015) was used to align sequencing reads to a reference genome (Stam *et al.*, 2019b). After alignment, featureCounts (Liao *et al.*, 2014) was used to identify the number of reads that mapped to genes. For featureCounts, all entries tagged as ‘gene’ were extracted from the gff annotation files and by adjusting the gene_id and transcript_id identifiers, this gene list was converted into a gtf annotation file. The downstream region of every gene was extended by 1 kb (the extension stops when it hits the next gene start site). featureCounts was modified to search for the tag ‘gene’ instead of the default ‘exon’ tag.

Differential gene expression analysis was carried out using the R package DESeq2 (Love *et al.*, 2014). DESeq2 uses the output of featureCounts to estimate the fold change in gene expression between different treatment groups. Default parameters from the DESeq2 package were applied and differentially expressed genes (DEGs) showing adjusted *P*-value <0.05 were considered significant.

Gene Ontology (GO) enrichment analysis was based on previously annotated ontologies (Stam *et al.*, 2019b). GO terms were selected for all candidate genes. The background frequency of each GO term is the number of genes annotated to that GO term in all genes, while sample frequency is the number of genes annotated to that GO term in the list of DEGs in this sample.

For the manual inspection of the functions of the differentially expressed overlapping gene candidates in laminarin- and *P. infestans-*treated samples, we performed a BLAST search, extracted gene names and functional annotation from the best hits, and if needed, performed a literature search for papers that described the functions of the described gene candidates (see 10.5281/zenodo.5101308).

### Gene expression analysis of the key indicators of phytohormone pathways by quantitative PCR

To independently evaluate phytohormone regulation in response to laminarin (1 mg ml^−1^) elicitation we tested the expression level of key indicators of three well-known defence phytohormones, *1-aminocyclopropane-1-carboxylic acid synthases 2* (*ACS2*) from the ET pathway (Gravino *et al.*, 2015), *isochorismate synthase* (*ICS*; Di *et al.*, 2017) and *phenylalanine ammonia-lyase* (*PAL*; Peng *et al.*, 2004) from the SA pathway, and *lipoxygenase D* (*LOXD*; Heitz *et al.*, 1997) from the JA pathway, in individual plant leaf discs of *S. chilense* upon treatment with laminarin and compared it with mock-treated (water) leaf discs. *Solanum chilense* reference names of the genes are provided in Supplementary Table S1.

Leaf discs of the plant LA1963-02 (chosen due to its high resistance observed in Kahlon *et al.* (2021a) were treated with laminarin, and MilliQ-H_2_O-treated leaf discs served as control. Experiments were performed on three different dates in three independent replicates each. Samples were treated for 1.5 h, snap-frozen in liquid nitrogen, and ground to a fine powder with a mortar and pestle. RNA was extracted using the Qiagen RNeasy plant mini kit according to the instruction manual. cDNA synthesis was performed using a Qiagen QunatiTect reverse transcriptase kit according to the instruction manual. Quantitative PCR (qPCR) was performed on the synthesized cDNA using Takyon Low ROX SYBR master mix ddTTP blue (Eurogentec Liège, Belgium). qPCR was performed in three technical replicates, and a non-template control was included. Primer pairs for each tested gene are indicated in Supplementary Table S1. *TIP-41* (Fischer *et al.*, 2013; Nosenko *et al.*, 2016) was used as a housekeeping gene for normalization, and primer efficiency was performed for all the primer pairs and is shown in Supplementary Table S1. The PCR reaction comprised 10 μl SYBR Green-ROX Mix, 0.3 μM forward primer and 0.3 μM reverse primer, and 3 μl cDNA with volume adjusted to 20 μl with MilliQ-H_2_O. The thermal cycling profile was set to a hot start at 95 °C for 3 min, followed by 40 cycles of amplification (95 °C for 30 s, 60 °C for 30 s, 72 °C for 1 min), 1 cycle melting (95 °C for 30 s, 65 °C for 30 s, 95 °C for 30 s), and at the end one cycle at 72 °C for 10 min. Melting curve temperatures were recorded at the end of the cycle for quality control. Data were evaluated with the software Agilent Aria MX 1.7, and relative gene expression was calculated following the method of Livak and Schmittgen (2001).

### ROS production measurement

To measure ROS production upon elicitation with laminarin, we performed a 96-well-plate assay based on chemiluminescence as described by Kahlon and Stam (2021b). Leaf discs from leaves of mature plants were made with a biopsy punch (4 mm, KAI Medical, Solingen, Germany) and incubated in a white 96-well flat-bottom plate overnight in 200 µl of 20 mM MOPS (pH 7.5) at room temperature. The next day buffer was removed and wells were supplemented with 75 µl HRP mix (10 µM horseradish peroxidase (HRP) and 10 µM L012). A baseline reading was performed for the initial 10 min using a Luminoskan Ascent microplate luminometer (Thermo Scientific) and then laminarin (1 mg ml^−1^ final concentration) dissolved in MOPS was added to the plate at 4 wells per plant. For each population, the assay was performed on three different dates with four technical replicates each for treatment and mock (MOPS) per date. In addition, we performed ROS production measurements with flg22 at a final concentration of 500 nM in population LA4330 (seven plants) to compare it with specificity of laminarin in ROS production. Normalization of data was performed by first averaging over the initial 6–10 min baseline reading, followed by normalization to the mock treatment for the treated leaf discs.

### Measurement of phytohormones and their derivatives

#### Measurement of ethylene

Leaf discs were obtained with a 4 mm biopsy punch and incubated overnight in Petri dishes containing milliQ-H_2_O at room temperature. Following overnight incubation, three leaf discs were added to glass vials (5 ml) containing 300 µl of milliQ-H_2_O. Laminarin was added in a final concentration of 1 mg ml^−1^ to three glass vials (samples) containing three leaf discs of one plant, and milliQ-H_2_O in three separate glass vials containing leaf discs for the same plant served as a negative control. Upon addition of elicitor or water, the glass vials were sealed with septa (Carl Roth GmbH). Samples were incubated for 3 h on a shaker at ~ 20–50 rpm (Heidolph Polymax 2040). Three hours post-incubation, 1 ml of air was retrieved from each samples with a syringe through the rubber cap and injected into a Varian 3300 gas chromatography machine containing an AlO_3_ column with length 1 m and 225 °C detector temperature, with 80 °C column and injector temperature. The gases used for the separation of ET from the sample were H_2_, N_2_, and O_2_ at 0.5 MPa each. The amount of ET was calculated based on the standard calculation as developed by Von Kruedener *et al.* (1995) using the area under the curve (AUC). In total, we measured up to nine samples per plant on three different dates, each date containing up to three samples.

#### Measurement of salicylic acid, jasmonic acid, abscisic acid, indoleacetic acid, phaseic acid, and dihydrophaseic acid

Samples for measurements of SA, JA, ABA, IAA, phaseic acid (PA) and dihydrophaseic acid (DPA) were also prepared based on the leaf disc treatment method; 150–200 leaf discs were made per plant using a 4 mm-diameter biopsy punch and incubated overnight in Petri dishes containing milliQ-H_2_O. The next day for each plant a six-well plate filled with milliQ-H_2_O was prepared for elicitation containing 25–30 leaf discs per well. Three wells were elicited with laminarin (1 mg ml^−1^) and in the remaining three wells milliQ-H_2_O was added as a control. The plates were incubated for 3 h on a shaker at ~20–50 rpm. Following the treatments, the leaf discs were transferred to 2 ml Eppendorf tubes, and residual water was pipetted out before snap-freezing the samples in liquid nitrogen.

Fine powder from the plant material was obtained after grinding the frozen leaf discs with a mortar and pestle in liquid nitrogen. The samples were then processed for extraction of the phytohormones and their derivatives as described by Chaudhary *et al.* (2020), with minor modifications. Ground material of 50–200 mg was transferred to a 2 ml bead beater tube (CKMix-2ml, Bertin Technologies, Montigny-le-Bretonneux, France). Twenty microlitres of internal standard solution containing indoleacetic acid-d2 (Sigma-Aldrich, Steinheim, Germany; 2.5 μg ml^−1^), salicylic acid-d4 (Olchemim, Olomouc, Czech Republic; 2.5 μg ml^−1^), (+)*cis*,*trans*-abscisic acid-d6 (Sigma-Aldrich; 2.5μg ml^−1^), and (−)*trans*-jasmonic acid-d5 (25μg ml^−1^; Santa Cruz Biotechnology, Dallas, TX, USA) were dissolved in acetonitrile and added to the samples and incubated for 30 min at room temperature. Following that 1 ml of ice-cold ethyl acetate (Merck, Darmstadt, Germany) was added to the samples and stored overnight at −20 °C. The next day samples were shaken for 3 × 20 s using the bead beater (Precellys Homogenizer, Bertin Technologies) at 6000 rpm with 40 s breaks in-between. The material was then filtered with a 0.45 μm pore size filter (Sartorius, Darmstadt, Germany) using a Minisart syringe. The filtrate was transferred to 2 ml tubes and vacuum dried. Then samples were reconstituted in 70 µl of acetonitrile and sonicated for 3 min. Two microlitres of the sample from the HPLC tubes (glass vials) was injected into the LC-MS/MS system. The MS method used measured positive and negative ionization mode within one run (polarity switching). Negative ions were detected at an ion spray voltage of −4500 V (ESI−) using ion source parameters: curtain gas (35 psi), temperature (550 °C), gas 1 (55 psi), gas 2 (65 psi), collision activated dissociation (−3 V), and entrance potential (−10 V). Positive ions were detected at an ion spray voltage at 4500 V (ESI+) using ion source parameters: curtain gas (35 psi), temperature (550 °C), gas 1 (55 psi), gas 2 (65 psi), collision activated dissociation (−3 V) and entrance potential (10 V) and 40 °C column oven temperature was at a QTRAP 6500+ mass spectrometer (Sciex, Darmstadt, Germany). MS/MS fragmentation was obtained and samples were separated by ExionLC UHPLC (Shimadzu Europa GmbH, Duisburg, Germany) using 100 × 2.1 mm^2^, 100 Å, 1.7 μm, Kinetex F5 column (Phenomenex, Aschaffenburg, Germany). Solvents used for separation were (A) 0.1% formic acid in water (v/v) and (B) 0.1% formic acid in acetonitrile (v/v) with a flow rate of 0.4 ml min^−1^. Chromatographic separation was performed with the gradient of 0% B for 2 min, increased in 1 min to 30% B and in 12 min to 30% B, increased in 0.5 min to 100% B, held 2 min isocratically at 100% B, decreased in 0.5 min to 0% B, and held for 3 min at 0% B. Phytohormone quantification was performed based on comparison with standard curves prepared with purified hormones and using the AUC. The final concentrations were obtained in nanograms of hormone per gram of fresh weight of the sample.

### Infection data

Data on *P. infestans* infections were taken from Kahlon *et al.* (2021a), using the same methods that were previously described in Stam *et al.* (2017). In these studies, detached leaves were drop-infected with a *P. infestans* solution (3000 sprongia ml^−1^). All leaflets of the compound *S. chilense* leaves were infected with a single drop and the infection frequency (IF) was calculated per leaf and summarized per plant and population. The data originate from the exact same plants as those used in this study.

### Validation of ethylene accumulation in delivering resistance in individuals from the southern coastal population

ET validation experiments were performed on two individuals (plant 05 and plant 10) from a southern coast population, LA4107, selected based on Pearson’s correlation among ET production and infection frequency. ET measurement in the leaf discs was performed as described above. ET blocking was performed by adding 5 µM aminoethoxyvinyl gylcine (AVG) (Sigma-Aldrich) to the samples, and 3 h post-treatment ET was measured by gas chromatography. The infection frequency upon treatment with 5µM AVG was determined using a detached leaf infection assay as described in Kahlon *et al.* (2021a): detached leaves were surface-sterilized with 70% ethanol and kept upside down (adaxial side facing upwards) in plastic boxes containing a wet tissue bed. The leaf set for AVG treatment was kept on a wet tissue bed made with water containing AVG (5 µM) and leaves were sprayed with 5 µM AVG following drop inoculation with *P. infestans* isolate Pi100 (3000 sporangia ml^−1^). The experiment was repeated on eight individual leaves per treatment. Throughout the experiment, 18–20°C temperature was maintained and boxes were kept in dark. The infection outcome was taken at 7 days post-inoculation.

### Statistical analysis

All the data analyses were performed in R software (version 3.4.4, R Core Team, 2020). ANOVA was performed with the function aov(), and post-hoc Tukey tests with the function TukeyHSD(), from the package stats. When the *P*-value was less than 0.05 it was considered significant. Pearson’s correlation was performed using the function cor(). The analyses were done for the 83 plants for which IF scores were available (Kahlon *et al.*, 2021a). Figures were made using the R package ggplot2.

## Results

### Laminarin elicits a transcriptional response overlapping with that induced by *Phytophthora infestans*

First, we set out to confirm whether the purified glucan elicitor laminarin can elicit oomycete-like early defence responses in *S. chilense*. Therefore, we infected *S. chilense* plants of population LA3111 with *P. infestans* or treated the plants with laminarin and measured the transcriptional response after 3 h.

**Fig. 1.**
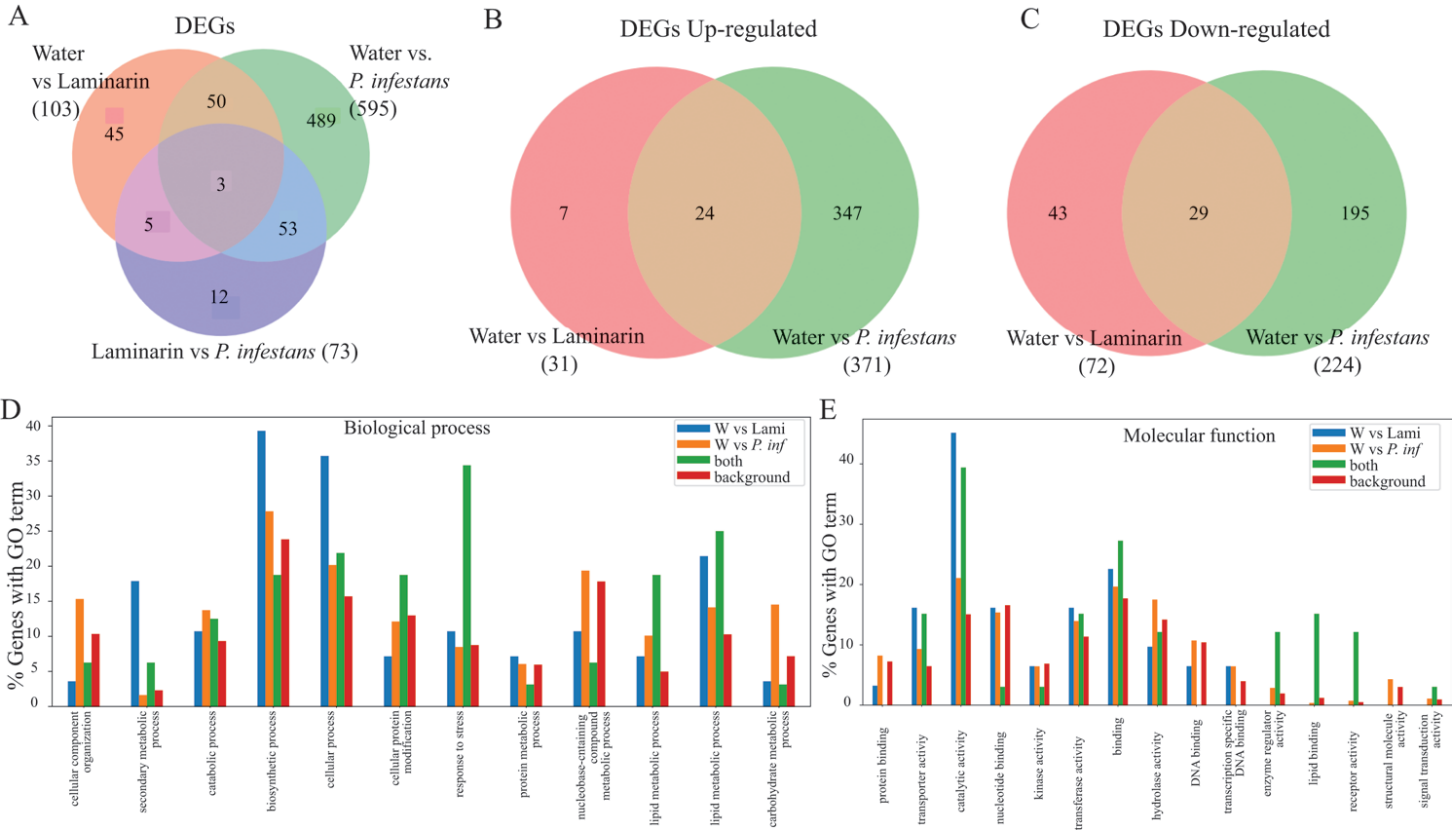
RNAseq analysis of the central population LA3111 (nine plants pooled per treatment) of *S. chilense* 3 h after *P. infestans* (3000 sporongia ml^−1^), laminarin (1 mg ml^−1^), and water treatment. (A–C) Differentially expressed genes (DEGs) overlap in different treatments: overall (A), up-regulated (B), and down-regulated (C). (D, E) Gene Ontology (GO) analysis of RNA-seq data showing percentage genes with signals for gene ontology terms for biological process (D) and molecular function (E) for laminarin versus *P. infestans* treatment.

As expected, infection with *P. infestans* triggered strong transcriptional responses. In total, we measured 595 DEGs (false discovery rate adjusted *P*-value <0.05) upon *P. infestans* infection (371 up-regulated and 224 down-regulated). Laminarin treatment resulted in 102 DEGs (31 up-regulated and 72 down-regulated) (Supplementary Table S2). More than 50% of the genes that were differentially expressed after laminarin treatment were overlapping with the *P. infestans-*associated response. For the up-regulated gene fraction, the overlap was 77%. Only a small number of DEGs (12) could be uniquely detected in a direct pairwise comparison between the Laminarin-treated and *Phytophthora*-treated samples, and thus the false positive discovery rate in this experiment is likely lower than 2% (Fig. 1A–C). To validate our hypothesis that laminarin triggers a decomplexified defence response, we analysed the annotations of the overlapping gene lists. Thirty-five percent of the overlapping genes are associated with the biological process ‘stress response’, and the overlapping fraction is significantly more often annotated with the GO terms ‘enzyme regulator activity’ and ‘receptor activity’ (chi-square test, *P*<0.01, Fig. 1D, E; Supplementary Tables S3, S4). Moreover, homologues of more than half of the overlapping genes are reported in the literature to be involved in defence responses (Supplementary Table S5). We found homologues of regulators of the plant ROS response (SOLCI005830700, Peroxidase *CEVI1*), key regulators of defence hormone signalling like *ER5* (SOLCI004643500, ET response); *LOX1* (SOLCI003764800, JA signalling) or *PAL3* (SOLCI000597200, SA signalling), and up-regulation of *Mitogen-activated protein kinases* (*MAPK*s, SOLCI002491100). This supported the view that laminarin can trigger a subset of oomycete-associated defence responses and is a suitable compound to study variation in basal defence responses in *S. chilense*.

To independently verify the involvement of laminarin-specific phytohormone-associated defence responses, we evaluated the expression of key regulators described in literature of three phytohormones, ET, SA, and JA, in an *S. chilense* individual from a different central population, LA1963 (plant 02). We observed an up to 7.8-fold increase in expression of *S. chilense ACS2* (SOLCI000989600), a key regular in the ET pathway in laminarin-treated samples as compared with water-treated samples (Supplementary Fig. S1). Next, we looked into key regulators from the two known pathways for SA regulation. We observed that *PAL*-like transcripts (SOLCI002546900) showed up to 14-fold increase in laminarin-treated samples as compared with water-treated controls. *ICS* (SOLCI004470400), the key regulator of the second SA pathway, was down-regulated (Supplementary Fig. S1). For the JA pathway, we performed qPCR on the *LOXD* (SOLCI003768300) gene and observed an up to 10-fold increase in the laminarin-treated samples (Supplementary Fig. S1). Thus, we confirmed differential regulation of key regulators in defence-associated phytohormone pathways.

### Reactive oxygen species production in *S. chilense* is highly polymorphic

**Fig. 2.**
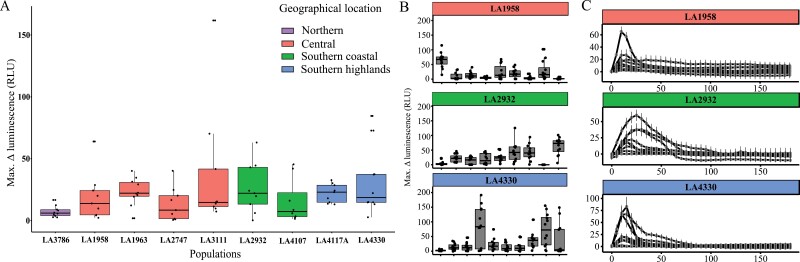
ROS accumulation in the leaf discs from *Solanum chilense* measured from 0 to 180 min after elicitation with laminarin (1 mg ml^−1^). (A) Overview for each of the populations. Each boxplot represents a populations, and each black dot is the mean measured value for one plant, obtained from three or four individual repetitions. Statistically significant pairwise comparisons between populations are shown in Supplementary Table S6. (B) Examples highlighting the within-population diversity for three of nine populations. Each boxplot represents an individual plant per population with data from one leaf disc represented as one data point accounting for up to 10–12 leaf discs per plant. Individual measurements were performed on three different dates (*n*=3–4 each date; 3 × 3 (4)=10 (12) leaf discs per plant). Each individual data point in (A) corresponds to the median of one of the boxplots shown in (B). Within-population pairwise comparison statistics are shown in Supplementary Table S7. (C) Differences in ROS kinetics for the three highlighted populations from (B). *x*-axis is minutes post-treatment; *y*-axis shows relative luminescence unit (RLU). Colours of the boxplots or header bars represent the geographical location of the population. Extended panels like (B) and (C) for all populations can be found in Supplementary Figs S3, S4. Each data point is a median similar to (B) and the error bars represent standard error to give a clear visualization of the different plants.

ROS is one of the important key regulators in basal immune responses, and regulators of the ROS pathway were ­differentially expressed in the RNAseq data (Supplementary Table S5). Therefore, we tested ROS production in 83 genetically distinct *S. chilense* plants upon elicitation with laminarin. Maximum ROS production upon laminarin elicitation was significantly different between the populations (Fig. 2A; Supplementary Table S6). The highest average ROS maximum was recorded in LA3111 and the lowest in LA3786.

When grouping the populations by geographical region and looking at the overall average in ROS maxima, the southern highlands group had the highest ROS production and the northern group had the lowest (Supplementary Fig. S2). We also found significant differences in ROS maxima within the individual populations for eight out of nine populations (Supplementary Table S7), with some plants showing high ROS production and others showing no detectable ROS production upon elicitation with laminarin (Fig. 2B; Supplementary Fig. S3).

We also observed variation in the kinetics of the ROS production, with plants in some populations not showing a clear single peak, but rather a longer-lasting ROS production. This phenomenon appeared more common and occurred most frequently in the southern populations (Fig. 2C; Supplementary Fig. S4).

To confirm the specificity of the observed ROS production towards laminarin, and to show that lack of observed ROS burst does not result from a general ROS signalling impairment in the plants, we further tested ROS production after elicitation with the bacterial PAMP peptide flg22 in all plants from population LA4330 (Supplementary Fig. S5). This revealed variable ROS production upon elicitation with flg22. Moreover, there appears to be no apparent correlation between the strengths of flg22- and laminarin-triggered responses. Some plants showed no flg22 response and a clear laminarin response or vice versa, and some plants showed responses of similar intensity. This suggests that the observed differences in some plants are elicitor-specific variation and not a general effect of ROS production ability or defence signalling pathways.

### Ethylene accumulation upon laminarin treatment is low in southern highland populations

**Fig. 3.**
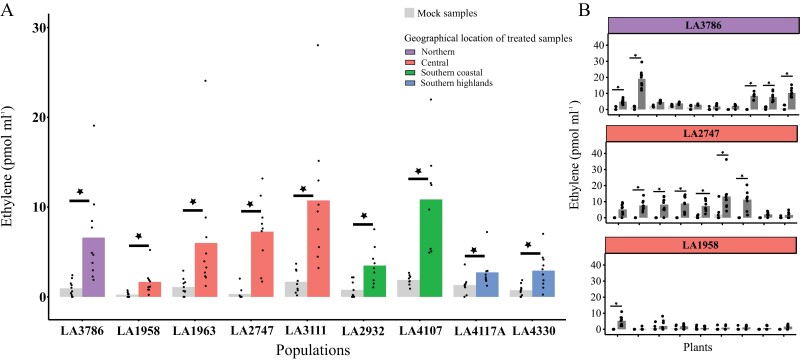
Ethylene (ET) accumulation in the leaf discs from *S. chilense* 3 h after elicitation with laminarin (1 mg ml^−1^) and mock (milliQ-H_2_O). (A) Each bar pair (light grey and coloured) represents an population. Each bar shows the mean of the population, and each dot represents the mean of one plant from three individual repetitions (as in B). (B) Each bar pair (light grey and dark grey) represents an individual from the population. Each bar shows mean of seven to nine data points that represent seven to nine sample measurements performed on three different dates (*n*=2–3 samples for each date), each sample containing three leaf discs. Significantly different ET accumulation in laminarin-treated samples from the mock-treated samples in an individual is represented by the star on the bar pair (ANOVA with post-hoc Tukey test on complete dataset). *y*-axis shows ET accumulation in pmol ml^−1^ headspace of the samples. Each panel in (B) shows a different population and colours represent the geographical location of the population. Panels for all additional populations can be found in Supplementary Fig. S6A. Statistically significant ET accumulation upon laminarin treatment is denoted with stars and other pairwise comparisons of within and between populations can be found in Supplementary Tables S9–S11.

To evaluate the role of phytohormones in the resistance differences observed in *S. chilense*, we first looked into ET production. Upon elicitation with laminarin, we observed that plants showed significant differences in ET production as compared with mock-treated samples (Fig. 3; Supplementary Tables S8, S9). Out of 83 plants tested, we found significantly induced ET production upon elicitation in 39 plants. Plant 02 from LA1963 showed a clear ET response, and so do several plants from population LA3111, confirming our RNAseq and gene expression qPCR results above (Supplementary Fig. S1; Supplementary Table S9). Hence, differential ET-pathway gene expression in these plants correlates with and might lead to laminarin-elicited ET accumulation.

We found significant differences in ET accumulation between the populations (Fig. 3A; Supplementary Table S10). Looking at the populations based on their geographical locations shows that the overall average of ET accumulation was lowest in the southern highlands group (Supplementary Fig. S2B). Within populations, we observed that the number of plants that significantly differ in ET response varied dependent on the population. Population LA3786 showed the most differences between individual plants and LA2747 was the most uniform, while population LA1958 showed nearly no ET response (Fig. 3B; Supplementary Table S11).

### Populations show high diversity in the basal level of phytohormones

Next, we looked into the production of two important defence-related phytohormones and their derivatives, for which we detected expression of key regulators in our RNAseq data, JA and SA, as well as other phytohormones and their derivatives that are known to be involved in stress responses: ABA, PA, DPA, and IAA.

**Fig. 4.**
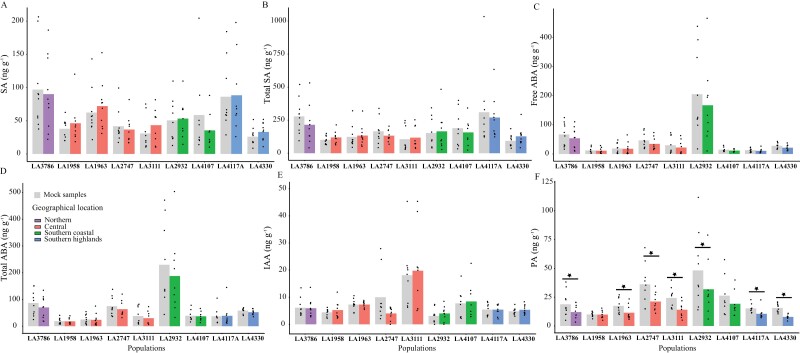
Phytohormone measures in the leaf discs from *S. chilense* 3 h after elicitation with laminarin (1 mg ml^−1^) and mock (milliQ-H_2_O). Each bar pair (light grey and coloured) represents a population. Each dot represents the mean of a single individual measured with at least three independent repetitions. Results for each individual plant can be found in Supplementary Fig. S6. *y*-axis shows phytohormone accumulation in ng g^−1^ of the samples. Colours represent the geographical location of the population. Statistically significant phytohormone accumulation upon laminarin treatment is denoted with stars and other pairwise comparisons of within and between populations can be found in Supplementary Tables S13–S17.

With our method, we were unable to detect quantifiable amounts of JA in any of the samples tested, but were able to quantify both free and total SA in the basal state and after elicitation. We observed that most populations did not show strong differences in the levels of SA (free and total) after elicitation with laminarin when compared with basal level of corresponding plants (Fig. 4A, B; Supplementary Fig. S6B, C; Supplementary Table S8). Four plants form an exception: two showed a higher amount of free SA (LA3111 plant 05 and LA4330 plant 05), and two showed a lower amount of free SA (LA2932 plant 12 and LA4107 plant 12) when compared with the mock-treated samples (Supplementary Fig. S6B).

Interestingly, we measured significant differences in basal levels of free and total SA within and between populations. The correlation coefficient between free and total SA content is 0.46 (*P*=1.10 × 10^−5^) (Supplementary Table S12), and therefore we treated free and total SA independently in further analyses. Both basal SA levels (free and total) were significantly different between the populations (Supplementary Table S13). As with ROS and ET responses, we also found significant differences within the populations for basal levels of both free SA and total SA content (Supplementary Table S14).

We also measured ABA, PA, DPA, and IAA, which are described as being important for biotic stress responses, and pathways of several of these hormones influence each other. We did not detect DPA in our samples. We detected basal levels for the phytohormones ABA (free ABA (Fig. 4C; Supplementary Fig. S6D) and total ABA (Fig. 4D; Supplementary Fig. S6E)), IAA (Fig. 4E; Supplementary Fig. S6F), and PA (Fig. 4F; Supplementary Fig. S6G). When evaluated on a complete dataset for laminarin treatment, no significant changes in the level of these phytohormones were observed except for PA (Supplementary Table S8). When significance was tested for individual plants, very few plants showed significantly different accumulation upon laminarin elicitation (Supplementary Fig. S6). For PA we observed a significantly lower amount after treatments when compared with the basal level in plants (Fig. 4F; Supplementary Fig. S6G; Supplementary Table S15), although in general basal levels of PA and PA levels upon elicitation were highly correlated (Pearson’s correlation coefficient of 0.74, *P*=2.2 × 10^−16^) (Supplementary Table S12). We observed a higher amount of basal levels of free and total ABA in LA2932 as compared with other populations, whereas IAA was higher in LA3111. The levels of all these phytohormones show significant differences between and within populations (Supplementary Tables S16 and S17, respectively) in an independent manner (Supplementary Table S12). Looking at the data of all the tested populations based on geographic location, we found higher levels of basal PA in the southern coast, IAA was higher in the central region, SA (free and total) was high in the north, and ABA levels were higher in northern and south coast populations (Supplementary Fig. S2).

### Multiple defence responses correlate with observed resistance phenotypes

To assess whether the individual defence responses measured in the plants can be associated with *S. chilense* resistance properties, we looked for correlations with previously generated data on the frequency with which *P. infestans* can infect *S. chilense* leaflets, the so-called infection frequency (IF) (Kahlon *et al.*, 2021a). We found no correlation between the observed ROS maxima and the IF observed with *P. infestans* (Pearson’s correlation coefficient of 0.09, *P*=0.37; Table 1), whereas we found a significant negative Pearson’s correlation of IF with ET accumulation (−0.36, *P*=0.0008; Table 1). The Pearson’s correlation of IF with basal levels of PA also showed a negative correlation (−0.2443413, *P*=0.026; Table 1), whereas we observed no strong or significant (*P*<0.05) correlation for SA, ABA, and IAA (Table 1).

**Table 1.** Pearson’s correlation of measured potential immunity-related factors at basal levels and upon elicitation with laminarin (1 mg ml^−1^) with the infection frequency (IF) of same plants upon inoculation with *P. infestans* Pi100 published in Kahlon *et al.* (2021a)

Parameter compared	*r*	*P*
IF–ROS	0.10	0.3748
IF–ET	**−0.36**	**0.0008**
IF–Free SA (basal)	−0.05	0.6290
IF–Total SA (basal)	−0.15	0.0690
IF–Free IAA (basal)	−0.13	0.2727
IF–Free ABA (basal)	−0.12	0.2737
IF–Total ABA (basal)	−0.12	0.2419
IF–PA (basal)	**−0.24**	**0.0260**

The correlation is shown for the all the measured components. Significant correlation is highlighted in bold. IF, infection frequency; *r*, Pearson’s correlation coefficient.

### The dominance of individual defence responses differs geographically

**Fig. 5.**
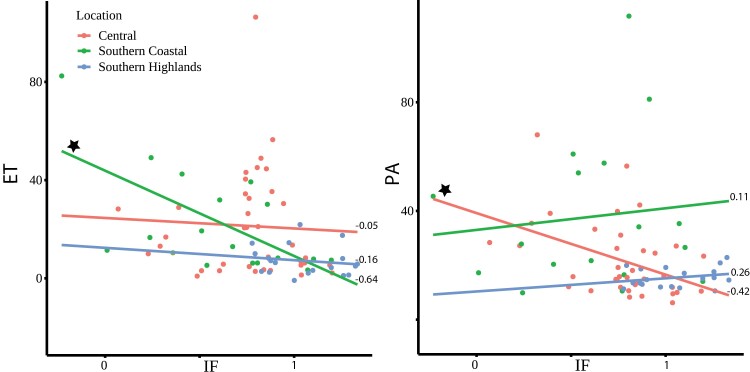
Correlations between the *P. infestans* infection frequency (IF, *x*-axis) and the measured Ethylene (ET) or phaseic acid (PA) phytohormone accumulation in ng g^−1^ sample or pmol ml^−1^, respectively (*y*-axis). Each dot represents the mean value of an individual plant from three individual repetitions. Infection frequencies were obtained from an independent experiment from the same plants as presented in Kahlon *et al.* (2021a). IF of zero indicates plants that are fully resistant and IF at 1 indicates 100% infection rate upon inoculation. Pearson’s correlation was calculated per geographic group. The star indicates significant correlations for the central and southern coastal populations for PA and ET, respectively.

All measured components showed geographical trends at basal and induced levels (Supplementary Fig. S2). This supports that the plants’ genotypes rather than the common experimental environment was the driver of metabolic differences between the plants. We hypothesize that different populations have adapted different defence strategies through adaptation to specific climatic niches. Thus, to confirm the possible larger effect of any of the measured components in certain geographical regions, we calculated the correlation coefficient for ET and PA for each of the geographical groups of *S. chilense*. We found that the effect of ET is most strongly correlated with resistance in the coastal populations, whereas PA showed the strongest correlation to resistance in the central group (Fig. 5; Supplementary Table S18).

### Ethylene plays a role in defence response in southern coast tested individuals

**Fig. 6.**
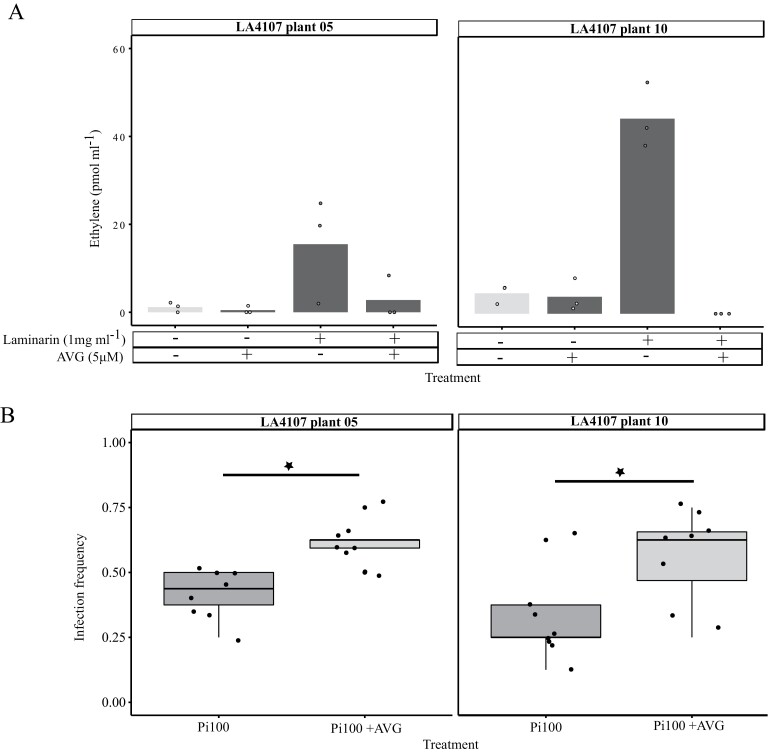
Ethylene (ET) inhibition assay on LA4107 plant 05 and plant 10. (A) ET accumulation in the leaf discs from *S. chilense* LA4107 plant 05 and 10, 3 h after elicitation with laminarin (1 mg ml^−1^), AVG (5µM), laminarin (1 mg ml^−1^) + AVG (5µM), and mock (milliQ-H_2_O). Light and dark grey pairs of bars represents plants with and without treatment with laminarin (1 mg ml^−1^), respectively. Each bar is the mean of three samples measured. *y*-axis shows ET accumulation in pmol ml^−1^ air of the samples. (B) Detached leaf infection assay of LA4107 plant 05 and 10 upon drop inoculation with *Phytopathora infestans* Pi100 (3000 sporangia ml^−1^) with and without AVG (5µM) treatment. *y*-axis represents infection frequency which is the ratio of infected leaflets divided by inoculated leaflets. Each dot represents the ratio from one leaf. Statistical significance is denoted by stars.

To confirm the contribution of ET to resistance in the coastal populations, we selected two plants from southern coastal population LA4107, one with relatively high ET production and another with relatively moderate ET production, with low and medium–high scores from the infection frequency spectrum, respectively. To verify the role of ET on the resistance outcome, we used AVG, a well-established chemical inhibitor, to halt the ET production in the selected plants and tested the ET accumulation after laminarin treatment. AVG was successfully able to inhibit the ET production up to 100% in the plant samples (Fig. 6A). After inoculation with *P. infestans* isolate Pi100, the plants indeed showed higher susceptibility when they were pre-treated with AVG as compared with control plants, confirming the positive role of ET in basal resistance in this population (Fig. 6B).

## Discussion

We previously used natural populations of *S. chilense* to show intraspecific variation in resistance against *P. infestans* (Kahlon *et al.*, 2021a). In this study, we evaluated several key components of basal defence responses in the same plants to explore molecular cues behind the previously observed phenotypic variation.

In order to reliably and reproducibly study defence components in this polymorphic plant species, and to rule out variation arising during the preparation of pathogen biological material, we used the glucan elicitor laminarin. Laminarin has been previously reported to activate basal immune responses such as ROS production, calcium influx, and MAPK activation in members of the *Solanaceae* family (Meénard *et al.*, 2004; Wanke *et al.*, 2020). We observed significant overlaps in DEGs in a *S. chilense* central population, LA3111, upon elicitation with laminarin and infection with *P. infestans* (Fig. 1). The majority of these genes are known for involvement in defence responses. We further showed differences in transcript levels via qPCR of key regulators of defence-related phytohormones after laminarin treatment in a plant of a different central population, LA1963. This suggests that laminarin can be used as a proxy for evaluating early basal immune response activation in *S. chilense.*

The RNAseq data of both *P. infestans* and laminarin treatments revealed regulation of homologues of several previously identified genes known in major defence pathways, like those of ROS production and both SA and JA signalling. These basal immunity components have been shown to be involved in *P. infestans* resistance in different solanaceous plant species. In cultivated tomato, reduced accumulation of ROS results in enhanced resistance against *P. infestans* (Cui *et al.*, 2016). Higher SA levels have a positive effect on *P. infestans* resistance in *S. tuberosum* (Halim *et al.*, 2007). Both, SA and ET contribute to resistance in *N. benthamiana* (Shibata *et al.*, 2010), and higher levels of JA and interplay with SA were observed in resistant cultivars of *Capsicum annuum* (Ueeda *et al.*, 2005). We observed high intraspecific diversity in the above-mentioned components early after elicitation with laminarin, as well as at basal levels. Large-scale intraspecific variation in basal immunity has also been reported on a transcriptional level in Arabidopsis accessions upon elicitation with the bacterial PAMP flg22 (Winkelmüller *et al.*, 2021).

Surprisingly, we did not find a strong correlation between the amount of ROS produced in a plant after elicitation with laminarin and its resistance properties (Fig. 2). ROS production upon biotic stress has often been considered a hallmark of successful recognition of pathogens and the activation of defence (Torres, 2010). ROS production linked to the perception of flg22 is often taken as an indicator for resistance against bacterial *Pseudomonas* spp. pathogens (e.g. in Arabidopsis (Smith and Heese, 2014) and in tomato (Roberts *et al.*, 2019)). Our study shows that laminarin-triggered ROS production in *S. chilense* cannot be used to estimate the basal resistance against *P. infestans*. Similarly, we also found no correlation between laminarin-triggered SA production or basal SA levels and *P. infestans* resistance (Fig. 4). Thus, these individual defence components triggered by laminarin either have a rather limited contribution to the observed *P. infestans* resistance in the populations, or ROS and SA are not directly involved in *P. infestans* resistance in *S. chilense*. On the other hand, laminarin has been shown to induce the ET pathway, but only sulfated laminarin (β-1,3-glucan sulfate) can induce the salicylic acid signalling pathway in *N. tabacum* and Arabidopsis (Meénard *et al.* 2004). In our RNAseq analysis, we do see more DEGs when plants are treated with *P. infestans* as compared with laminarin. In the future, it would be interesting to evaluate the effects of sulfated laminarin and other known PAMPs from *P. infestans* in order to dissect the defence responses further.

Our data do support that resistance observed in our plant species can be correlated to different components in the plants: induced ET (Fig. 3) and also basal levels of the phytohormone PA (Fig. 4). This is in line with the hypothesis that basal defence is regulated by a complex network of interacting components from plants and pathogens (Windram and Denbi, 2015; Kahlon and Stam, 2021a). We also observed that the strength of the correlation is dependent on the geographical region from which the plants originated (Fig. 5). Calculations per ­population would be even more interesting, but due to the limited number of plants per population, these calculations would lack statistical power.

It can be assumed that in each population multiple components play important roles, but that due to sample size limitations, these effects were not picked up. The generation of generalized linear mixed models, testing the combined effect of multiple components, would be desirable in this context, though this would require a lot of additional data.

It has previously been shown that ROS production leads to SA production in a positive feedback loop in defence responses in Arabidopsis (reviewed by Herrera-Vásquez *et al.*, 2015). We did not observe such a correlation among ROS production and SA production at early time points, nor did we observe a correlation between SA and ET production as observed in tomato resistance against the fungal pathogen *Fusarium oxysporum* (Di *et al.*, 2017). Interestingly, the suppression of ABA biosynthesis and activation of ET biosynthesis upon copper ion treatment enhances resistance against *P. infestans* in potato seedlings (H. Liu *et al.*, 2020), whereas in our system ET positively contributed to resistance observed against *P. infestans*.

In our assays, ET had the strongest role in early defence. ET has previously been described in association with various defence responses. In Arabidopsis, the Resistance to Powdery Mildew 8 (RPW8)-mediated defence response is regulated by an ET-mediated feedback loop (Zhao *et al.*, 2021). For solaneceous species, the activation of defence-related genes in *P. infestans*-resistant potato cultivars upon exogenous ET treatment has also been reported in a recent transcriptome study by Yang *et al.* (2020), and laminarin triggers the expression of ET-dependent defence genes in *N. tabacum* (Meénard *et al.*, 2004). A positive role of ET production has been reported in relation to resistance to *C. fulvum* in tomato plants carrying corresponding resistance genes against a specific *C. fulvum* race (Hammond-Kosack *et al.*, 1996). Another study showed that ET is also involved in resistance to the fungal pathogen *B. cinerea* and certain wound responses in tomatoes, with no clear role of JA or SA observed (Díaz *et al.*, 2002).

We further showed geographical variation in basal and induced levels of each component and expect that the role of each component might differ between populations due to variation in habitats. Interestingly, the role of ET was stronger in the coastal populations and experimentally verified with ET inhibition assays in plants from coastal population LA4107 (Fig. 6). The stronger association of ET and resistance specifically in the southern coastal populations could be a result of specific adaptation processes in these populations. This could potentially reflect an added benefit of the development of stronger ET signalling in these populations as a result of specific habitat adaptation, e.g. to deal with potential abiotic stresses, like salt or temperature. General temperature dependency of defence regulation and the involvement of phytohormone signalling has been shown for both cold (Wang *et al.*, 2019) and heat stress (Huang *et al.*, 2021). ET has been shown to be a crucial phytohormone when it comes to coping with salinity stress in plants (Riyazuddin *et al.*, 2020). The positive effects of ET in salt tolerance have been illustrated in Arabidopsis (Yang *et al.*, 2013) and *Zea mays* (Freitas *et al.*, 2018). In a study by Kashyap *et al.* (2020), *S. chilense* plants under salt stress coped better than cultivated *S. lycopersicum* due to a better anti-oxidant system. We also observed high basal levels of the phytohormone ABA in the coastal population LA2932 (Fig. 4C, D). ABA is highlighted to be an important phytohormone for abiotic stress tolerance including salinity stress (reviewed by Zhu, 2002; Ng *et al.*, 2014).

The genotype-to-phenotype linkages in systems biology are complex. In a diverse panel of wild and domesticated tomatoes, basal resistance against the generalist fungal pathogen *B. cinerea* has been reported to be dependent on the interaction of multiple loci among both host and pathogen (Soltis *et al.*, 2019). Here we presented a decomplexification approach, where more components can be added to understand both the triggers (by testing different elicitors) and the outcomes (by measuring more responses). As highlighted by Marshall-Colón and Kliebenstein (2019), such future studies should be performed using large-scale metabolomics and transcriptomics analyses, to determine the key regulators underlying the measured responses and to be able to appreciate the intrinsic value of the complexity of signalling networks.

In our previous studies (Stam *et al.*, 2017; Kahlon *et al.*, 2021a) we showed no strong signs of host adaptation towards resistance to *P. infestans*, as resistance shows no clear geographical pattern of adaptation. However, our current results indicate that different coping mechanisms are present in each of these populations, possibly due to specific adaptation to the niches that the plants inhabit in each region. Examples of such specific adaptations have also been observed for other host–parasite interactions. Populations of *Eruca vesicaria* (syns. *Eruca sativa*, wild rocket) from Mediterranean and desert habitats showed activation in defence responses via two different mechanisms when challenged with the generalist herbivore *Spodoptera littoralis*. Mediterranean plants showed accumulation of glucosinolates and desert plants showed induced levels of a specific protease inhibitor (Ogran *et al.*, 2016). Bevan *et al.* (1993) showed immense differences in phenotypes of two populations of *Senecio vulgaris* (groundsel) against *Erysiphe fischeri* and proposed different populations have evolved different survival strategies against the same pathogen. In Arabidopsis, combined effects of genetic variation and differences in environmental factors also shape defence-associated metabolite contents (Katz *et al.* 2021).

Together our data support high complexity of *S. chilense*’s defence response to the general glucan elicitor laminarin. These responses might contribute to *P. infestans* resistance by additive or network functions. At single geographic locations, certain plant hormones play a bigger role than at others. We hypothesize that wild plants adapt to the local abiotic environment, and hormones may be key to this adaptation. The corresponding defence machinery might simultaneously undergo co-adaptation to cope with biotic stress. We speculate that plants’ high connectivity between abiotic and biotic signalling results in the necessity to habitat-specifically recruit different defence pathways and that the nature of the involved hormones accordingly differs in the wild. This would be determined not only by the local pathogens but also strongly by the abiotic environment and highlights the need for further population-scale studies on pathogen resistance mechanisms (Kahlon and Stam, 2021a, b).

## Supplementary data

The following supplementary data are available at *JXB* online.

Fig. S1. Expression levels of key defence regulators.

Fig. S2. Geographical grouping for the measured defence components.

Fig. S3. Maximum ROS accumulation in the leaf discs from *Solanum chilense* plants.

Fig. S4. Kinetics of ROS production of *S. chilense* plants upon laminarin treatment.

Fig. S5. Kinetics of ROS production of *S. chilense* population LA4330 with different elicitor treatments.

Fig. S6. Phytohormone levels upon laminarin treatment in individual *S chilense* plants.

Table S1. Details of gene IDs and primer pairs used for gene expression analysis via qPCR for *Solanum chilense* population LA1963 plant 02 upon elicitation with laminarin (1 mg ml^−1^).

Table S2. List of differentially expressed genes in plants of central population LA3111 of *S. chilense* upon *P. infestans* inoculation and laminarin treatment.

Table S3. Chi-square test for Molecular Function overlap set versus all DEGs.

Table S4. Chi-square test for Biological Process overlap set versus all DEGs.

Table S5. List of overlapping differentially expressed genes in plants (LA3111) treated with *P. infestans* and laminarin.

Table S6. Pairwise comparisons of ROS maxima observed upon elicitation with laminarin in *S. chilense* populations.

Table S7. Pairwise comparisons of ROS maxima of plants within different populations of *S. chilense* upon elicitation with laminarin.

Table S8. Statistical significance of increase in production of different phytohormones calculated using ANOVA for 83 plants of *S. chilense*.

Table S9. Level of ET upon laminarin elicitation tested for significant increase in all the tested plants, showing statistically significant plants.

Table S10. ET accumulation comparison between population of *S. chilense* showing statistically significant pairwise comparisons (calculated using ANOVA, with post-hoc Tukey test) of populations upon elicitation with laminarin.

Table S11. Pairwise comparisons of ET accumulation in plants of *S. chilense* within a population performed using ANOVA with post-hoc Tukey test showing statistically significant (*P*<0.05) groups.

Table S12. Pearson’s correlation calculated for comparing different phytohormones (free and total basal levels) and comparison of correlation of different phytohormone level of plants.

Table S13. Comparison of basal levels of free and total SA between populations of *S. chilense* along with significantly different groups (ANOVA with post-hoc Tukey test *P*<0.05).

Table S14. Pairwise comparisons of basal levels of free and total SA within populations of *S. chilense* and significantly different groups calculated using ANOVA with post-hoc Tukey test (*P*<0.05).

Table S15. PA basal and induced (upon elicitation with laminarin) levels compared in 83 tested plants showing plants with significantly different levels upon treatment.

Table S16. Basal level of phytohormones PA, ABA (free and total), and IAA, between the populations tested for significant differences, showing pairwise comparisons that were significantly different calculated using ANOVA with post-hoc Tukey test.

Table S17. Basal level of phytohormones PA, ABA (free and total), and IAA within-population differences of *S. chilense* tested using ANOVA with post-hoc Tukey test showing significant pairwise comparisons (*P*<0.05).

Table S18. Pearson’s correlation calculated for ET accumulation upon laminarin elicitation and PA basal level with the infection frequency of same plants upon inoculation with *P. infestans* Pi100 published in Kahlon *et al.* (2021a) after grouping the plants based on their geographic origin.

erad087_suppl_Supplementary_Figures

erad087_suppl_Supplementary_Tables

## Data Availability

Analytical results are included in the supplementary data files. Raw sequence data (Illumina reads) are uploaded to NCBI SRA and available under PRJNA746795. All scripts used for the analyses were adapted from and can be found in Kahlon *et al.* (2021a). Raw data from all analytical measurements, as well as all intermediate data files (e.g. FeatureCount output files), are available at Zenodo https://doi.org/10.5281/zenodo.5101308; (Kahlon *et al.*, 2021b).
